# Detailed Analysis of Responses from Older Adults through Natural Speech: Comparison of Questions by AI Agents and Humans

**DOI:** 10.3390/ijerph21091170

**Published:** 2024-09-03

**Authors:** Toshiharu Igarashi, Katsuya Iijima, Kunio Nitta, Yu Chen

**Affiliations:** 1 Simulation of Complex Systems Lab, Department of Human and Engineered Environmental Studies, Graduate School of Frontier Sciences, The University of Tokyo, Tokyo 277-8563, Japan; chen@edu.k.u-tokyo.ac.jp; 2 AI-UX Design Research Institution, Advanced Institute of Industrial Technology, 10-40 Higashi-Oi 1-Chome, Shinagawa, Tokyo 140-0011, Japan; 3 Institute of Gerontology (IOG), The University of Tokyo, Tokyo 113-8656, Japan; peilu-x@aiit.ac.jp; 4 Institute for Future Initiatives (IFI), The University of Tokyo, Tokyo 113-0033, Japan; 5 Tsukushikai Medical Corporation, Tokyo 186-0005, Japan; nitta.kunio.medical@gmail.com

**Keywords:** AI agents, cognitive function estimation, Alzheimer’s disease, dementia, psychological burden

## Abstract

In recent years, an increasing number of studies have begun to use conversational data in spontaneous speech to estimate cognitive function in older people. The providers of spontaneous speech with older people used to be physicians and licensed psychologists, but it is now possible to have conversations with fully automatic AI agents. However, it has not yet been clarified what differences exist in conversational communication with older people when the examiner is either a human or an AI agent. In this study, elderly people living in the community and attending a silver human resource center and a day service center were the subjects. Dialogues were conducted using generic interview items for estimating cognitive function through daily conversation, which were developed through research on estimation methods for cognitive function. From the data obtained from the dialogues, we compared the effects of human–AI interaction on the number of utterances, speaking time, and silence time. This study was conducted at a facility in Japan and included 32 subjects (12 males and 20 females). The results showed significant differences between human and AI dialogue in the number of utterances and silent time. This study suggests the effectiveness of AI in communication with older people and explores the possibility of using AI in social welfare.

## 1. Introduction

In modern societies, including Japan, the rapid aging of the population poses a significant challenge. As of 2020, elderly individuals aged 65 and above comprise about 28% of the total population in Japan, and this proportion is expected to exceed 30% by 2030 [1]. The demographic shift towards an older population significantly impacts healthcare, welfare, and economic activities. Social isolation, which worsens as society ages, is a critical issue. Many elderly individuals experience reduced contact with family and friends, leading to increased feelings of loneliness, which studies have shown to adversely affect psychological and physical health [2]. This is partly due to a lack of social support.

Moreover, cognitive decline in the elderly is a significant concern. Aging is commonly associated with reductions in memory and cognitive abilities [3], which can impair independence and degrade the quality of life for the elderly. However, recent research indicates that appropriate social and cognitive stimuli can maintain or even improve cognitive functions [4], particularly suggesting that dialogue and social activities could reduce the risk of dementia.

To address social isolation and cognitive decline, the use of artificial intelligence (AI) is gaining attention. With advancements in AI technology, new possibilities for elderly support are being explored. AI can complement and sometimes substitute traditional caregiving and support methods. For instance, AI-driven communication support systems can potentially reduce social isolation among the elderly. These systems utilize speech recognition and natural language processing technologies to facilitate conversations with the elderly [5]. Additionally, communications facilitated through AI-equipped robots or virtual agents can provide the elderly with opportunities for regular dialogue and information exchange [6].

Furthermore, AI could also aid in maintaining and enhancing the cognitive functions of the elderly. By integrating AI into cognitive training programs, customized interventions suitable for individual cognitive states can be developed, which are potentially effective in preventing dementia [7]. AI is also used for the early detection of cognitive impairments through automated speech analysis and digital voice evaluation [8,9]. Moreover, AI excels in analyzing large data sets and recognizing patterns, contributing to elderly health management and medical predictions [10].

As research and development progress, the role of AI in supporting the elderly is expected to grow significantly. Utilizing AI’s capabilities and advancing human-centered design could improve the quality of life for the elderly. Silver human resource centers and day service centers play crucial roles in enabling elderly social participation and maintaining physical and mental health through interactions with others. However, the impact of the quality and frequency of dialogues in these settings on the psychological health of the elderly is not yet fully understood.

This study investigates the differences in the number of utterances, speech duration, and silence times in dialogues conducted with elderly individuals from silver human resource centers and day service centers, comparing interactions with humans and AI. Cognitive functions were also measured using the MMSE. These validations examined the impact of AI-facilitated dialogues on the elderly, suggesting that AI-mediated communication could effectively ameliorate social isolation among the elderly.

## 2. Related Work

### 2.1. Experiments with Communication Robots

Communication robots and smart home systems and health monitoring technologies for the elderly have attracted much attention in recent years. These technologies aim to support older adults to live in their own homes, increase independence, and improve healthcare outcomes [11,12]. Asgharian et al. (2022) summarized studies on mobile service robots used for elderly care since 1999 and pointed out that robots can support daily activities, such as reminders, housework, and health monitoring, which can improve the welfare of the elderly and reduce the burden on caregivers [13]. Igarashi et al. (2022) pointed out the need for appropriate technology to help the elderly live independently as long as possible and developed a self-disclosure function to facilitate continuous interaction with the robot. Their experiments demonstrated that this feature effectively increased the quantity and quality of verbal interactions with the elderly [14]. In addition, NEC’s Papero-i-based dementia early detection system, which performs cognitive assessment based on natural conversations with elderly people living alone and notifies family members via social media, has also shown effectiveness [15,16]. On the other hand, challenges still remain in the implementation of these technologies, including user acceptance, privacy concerns, and the need for customized solutions tailored to different elderly populations [12].

Several factors influence older adults’ acceptance of technology, including perceived benefits, ease of use, social support, and individual characteristics [17,18]. Future research will focus on mobile devices and their impact on the quality of life of the elderly [19] and the need to adopt an integrated approach that takes into account both technological characteristics and the social context of use [18]. This study may provide one suggestion in examining the impact of AI agent technology on the quality of life of the elderly.

### 2.2. Virtual Agent Dialogue Experiments

Conversational virtual agents, used as avatars with human-like or other visual appearances, conduct dialogues with humans across mobile, web, and audio-based platforms using machine learning and natural language processing. International studies, including one from the UK, have used pre-recorded questions to engage virtual agents in dialogue with groups of individuals with mild cognitive impairments and healthy controls, revealing shorter speech durations among the impaired group. This suggests the potential of voice analysis in diagnosing cognitive impairments [20]. In Japan, NTT Communications Corporation launched a “Brain Health Check Dial” service in 2022, allowing users to anonymously check brain health based on a short speech sample and voice quality analyzed by AI [21].

In Japan, NTT Communications Corporation launched the “Brain Health Check Dial” service in 2022. This service uses a 20 s speech sample and voice quality to allow AI to assess changes in cognitive function, enabling users to anonymously check their brain health [22]. Experiments targeting patients with cognitive impairments demonstrated that conversations with agents are feasible even for those with cognitive disabilities. The agents were well-received, and providing interesting topics led to increased speech output [23].

Furthermore, studies evaluating the impact of AI-based dialogue agents on mental health and welfare have shown effectiveness in reducing the symptoms of depression and distress, though no significant improvements in overall psychological well-being were observed. The user experience was significantly influenced by the therapeutic relationship between humans and AI, engagement with the content, and effective communication [24]. A 14-day user study with 19 participants showed that while elderly users mainly used agents for music appreciation and reported high satisfaction, younger individuals often used them to enhance everyday efficiency [25]. Additional research has developed voice-controlled smart home systems for the elderly, confirming that conversational agents provide natural interactions and are useful in supporting daily life. These agents have been noted to potentially reduce feelings of social isolation and provide cognitive support [26]. Studies have also analyzed barriers and facilitators for elderly usage of voice assistants, indicating that improvements in usability can increase their adoption [27]. Meanwhile, discussions on providing interactive and responsive interventions for mental health and psychological well-being are increasingly incorporating the field of robotics, although empirical evidence and the utility of using Socially Assistive Robots (SARs) in mental health interventions are still largely unknown [28].

This study’s significance lies in clarifying the differences in speech duration and the number of utterances in dialogues with AI versus humans, targeting both cognitively high-functioning and low-functioning elderly individuals. There is a scarcity of direct comparisons between human and AI dialogues in the literature, especially regarding the number of utterances, speech duration, and silence times. By examining these aspects, this research aims to deepen our understanding of how AI agents affect elderly communication behaviors and contribute to the design of dialogue interfaces tailored to cognitive states.

### 2.3. Correlation between Speech and Cognitive Function in the Elderly

Multiple studies have indicated a relationship between speech characteristics, such as speech duration, number of utterances, and silences, and both dementia and quality of life in elderly individuals. Fukaya (2016) noted that speech duration is significantly shorter for elderly residents in nursing homes, especially for those who are bedridden [29]. Schröder (2010) observed that non-verbal communication abilities can last longer than verbal abilities in dementia patients, and that there is a complex relationship between communication ability and the quality of life [30]. Lee (2011) found that patients with Alzheimer’s disease often exhibit pauses and prolongations during speech, which diminishes both the productivity and effectiveness of their verbal expression [31]. These studies suggest a link between speech characteristics and dementia, but there has been insufficient research on speech characteristics in the elderly using AI agents.

### 2.4. Contribution

Although a fundamental cure for dementia has not yet been found, drugs have been developed to slow the progression of the disease. Therefore, early detection of cognitive decline is a crucial issue, and in recent years, the use of artificial intelligence (AI) in detecting cognitive decline has attracted attention.

For instance, AI is being utilized for the early detection of cognitive impairments through automatic speech analysis and digital voice evaluation [8,9]. Recent studies have also revealed that dementia groups can be classified into three categories—moderate, mild, and MCI—based on daily conversations [32].

Furthermore, AI can complement and sometimes replace traditional caregiving and support methods. AI’s ability to analyze large datasets and recognize patterns contributes to elderly health management and medical predictions, opening new possibilities for elderly support [10]. For example, AI-based communication support systems could potentially reduce social isolation among the elderly. These systems use speech recognition and natural language processing technologies to facilitate conversations with the elderly [5]. Additionally, promoting communication through AI-equipped robots or virtual agents can provide the elderly with regular opportunities for dialogue and information exchange [6].

Such AI-driven conversations may also serve as a form of training for elderly individuals living alone in the community, potentially helping to maintain cognitive functions. By integrating AI into cognitive training programs, customized interventions tailored to individual cognitive states can be developed, potentially serving as a preventative measure against dementia [7]. Therefore, leveraging AI’s capabilities and advancing human-centered design can enhance the quality of life for the elderly. However, there is still a lack of understanding of what occurs during conversations between elderly individuals and AI.

This study conducts a detailed analysis using semi-structured interview questions capable of estimating cognitive function to elucidate why these items can categorize cognitive function. This study targeted elderly individuals living independently in the community, not residing in facilities, and conducted a detailed analysis of the number of utterances, speech duration, and silence time in conversations. Two types of conversation partners were prepared for the elderly: (a) a human and (b) an AI agent on screen. The same set of questions was administered to both types of partner a month apart. Additionally, the participants were divided into high and low cognitive function groups, and the differences between the two groups were analyzed. This research provides significant insights into the nature of conversations with humans and AI among the elderly.

## 3. Methods

### 3.1. Experimental Design

Participants underwent three assessments: a cognitive function test using the Mini-Mental State Examination (MMSE), routine conversations with humans, and conversations mediated by AI. The MMSE used can be administered in 6–10 min and consists of 11 items that assess orientation, memory, calculation ability, language abilities, and visuospatial skills, with a total score of 30 points. A score below 23 suggests possible dementia, and a score below 27 suggests mild cognitive impairment (MCI) [33]. The Japanese version of the MMSE (MMSE-J) was utilized in this study [34]. For the conversational tests, generic hearing items developed by Igarashi et al. (2023) for estimating cognitive function through routine conversation were adopted [35]. These items were designed to assess family history, physical condition, interests, daily activities, and cognitive abilities related to memory and orientation. They were validated by five certified psychologists working in hospitals. These items were further refined for this study, categorizing them into six areas: temporal orientation, spatial orientation, family history, daily activities, physical condition, and interests, with a total of 30 questions designed for both AI and human conversations (Table 1). Conversations based on these questions were conducted over a month apart. This study was conducted with the approval of the ethical review by the University of Tokyo and Tokyo Metropolitan Institute of Technology (code: 23009, 19 October 2023).

### 3.2. Participants

According to the definition by the World Health Organization (WHO), elderly people are individuals aged 65 years and older. In Japan, the Ministry of Health, Labor and Welfare also references the WHO definition in its resources. Additionally, the Statistics Bureau of Japan classifies individuals aged 65 and above as elderly. Based on this, the present study also defined elderly people as those aged 65 and older. This study was conducted at a senior talent center and a day service center in Tokyo, involving 32 elderly participants (12 males and 20 females). The senior talent center refers to a resource center for individuals aged 65 and over who have retired from work but still possess a willingness to work, offering opportunities for temporary jobs through mediation. The day service center is a facility providing day services specifically for dementia patients. It serves elderly individuals whose cognitive functions are declining but do not require long-term institutionalization, offering a place for them to prevent further cognitive decline and engage in communication with others. The participants were elderly people living in the community who were not long-term residents of institutions or hospitals. In addition, to avoid the influence of dialect, participants were residents of Tokyo. Furthermore, this study was limited to those who had no physical ailments such as hearing loss and had no difficulty in listening to and speaking in general conversation. Participants were informed of the purpose and content of this study and their consent was obtained. For elderly people with cognitive decline, this study was explained to their family members and their consent was obtained.

### 3.3. Data Collection

The number of utterances, speech duration, and silence periods were recorded for both AI-mediated and human conversations, allowing for quantitative analysis of the interaction quality and the elderly’s responses. The content of the conversations was transcribed by a professional service.

Several modules were used to implement the AI agent, an avatar with AI dialogue capabilities. First, the author modeled the AI agent using VRoid Studio [36] to design it as if it were having an interpersonal conversation. For displaying the 3D character in a browser, @pixiv/three-vrm [37] was used. @pixiv/three-vrm is a library for loading and displaying VRM, a format for handling humanoid 3D avatar model data using three.js, on a browser, and is open-sourced by Pixiv. The background of the character incorporates the background of the room in the facility where the dialogue is taking place, creating an environment in which the user seems to be conversing with a real person.

**Figure 1:**
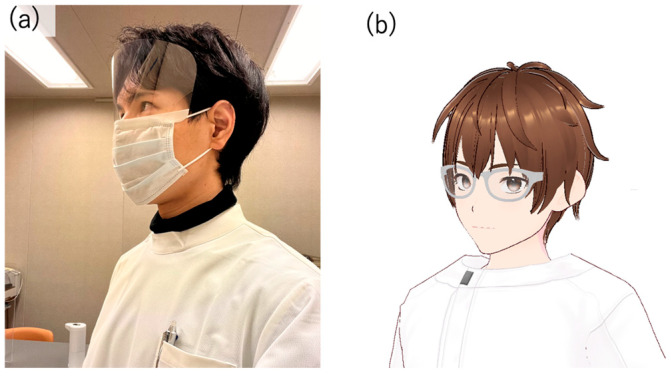
(**a**) The appearance of the author who conducted the interpersonal conversation and (**b**) the appearance of the AI agent modeled based on the author’s appearance.

In this study, the author executed a conversation with a human being and designed the AI avatar to resemble the appearance of a person (Figure 1). The aim was to minimize the influence of the avatar’s appearance on the test results, even though there are individual differences in impressions of appearance, because using an avatar whose appearance is significantly different from the person the participants assumes would be speaking may affect the test results. The Web Speech API (Speech Recognition) [38] was used for user speech recognition. Since speech recognition can be difficult when the timing of the agent’s speech and the participant’s speech overlap, we implemented a system in which the participants wear a pin microphone and speech is recognized only while a button is pressed. Responses from the system were generated using the Koeiro API [39], and the AI agent’s mouth was designed to move in sync with the speech using lip-sync functionality. The quantitative data obtained was analyzed using Microsoft^®^ Excel^®^ 2021 MSO.

### 3.4. Protocols in Conversation Design

**Figure 2:**
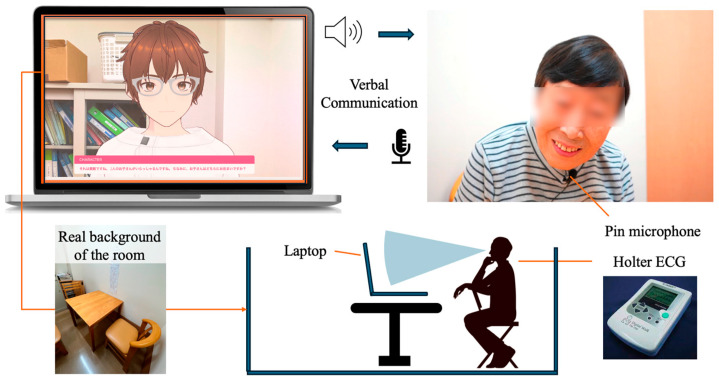
Protocol of daily conversation system for cognitive function estimation by AI agents.

**Figure 3:**
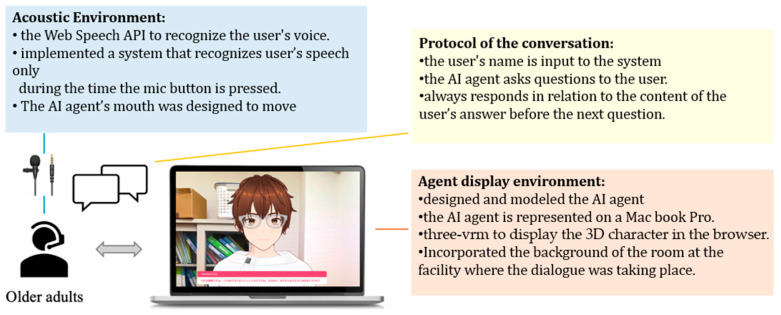
Configuration diagram showing the entire system.

The conversation design protocol was created using a semi-structured interview technique with fixed question content. The conversational protocol was designed to ensure that the completion of a conversation about a particular question is clear, with the AI agent always responding to the user’s answer with a response related to its content before moving on to the next question. Only the portion of the response corresponding to the user’s answer was implemented using the ChatGPT API [40] (Figure 2 and Figure 3).

## 4. Results

The study participants comprised 32 elderly individuals (12 males and 20 females) living in the community. Participants from the senior talent center included nine males and six females, with an average age of 74.73 years (SD = 5.48) and an average MMSE score of 28.67. The MMSE scores for participants from the senior talent center ranged from a maximum of 30 to a minimum of 26.

Participants from the day service center included 3 males and 14 females, with an average age of 82.59 years (SD = 4.96) and an average MMSE score of 13.47. The MMSE scores for participants from the day service center ranged from a maximum of 25 to a minimum of 7. Participants were categorized into high and low cognitive function groups based on an MMSE score cutoff of 25.

### 4.1. Conversations with Humans or AI

For conversations with a human interlocutor, the overall average number of utterances was 113.91, the average speech duration was 32.88 s, and the average silence duration was 4.19 s (Table 2). Specifically, for participants from the senior talent center, the average number of utterances was 123.96, the average speech duration was 42.56 s, and the average silence duration was 4.16 s. For participants from the day service center, the average number of utterances was 104.87, the average speech duration was 24.24 s, and the average silence duration was 4.21 s.

In conversations with the AI, the overall average number of utterances was 59.55, the average speech duration was 17.25 s, and the average silence duration was 5.01 s. Specifically, for participants from the senior talent center, the average number of utterances was 21.86, the average speech duration was 10.22 s, and the average silence duration was 3.76 s. For the participants from the day service center, the average number of utterances was 92.58, the average speech duration was 23.42 s, and the average silence duration was 5.34 s.

### 4.2. Comparison between Senior Talent Center and Day Service Center

For participants from the senior talent center, the overall average number of utterances was 73.76, the average speech duration was 26.65 s, and the average silence duration was 4.04 s. In human conversations, the average number of utterances was 123.96, the average speech duration was 42.56 s, and the average silence duration was 4.16 s. In AI conversations, the average number of utterances was 21.86, the average speech duration was 10.22 s, and the average silence duration was 3.76 s.

For participants from the day service center, the overall average number of utterances was 98.58, the average speech duration was 23.83 s, and the average silence duration was 4.86 s. In human conversations, the average number of utterances was 104.87, the average speech duration was 24.24 s, and the average silence duration was 4.21 s. In AI conversations, the average number of utterances was 92.58, the average speech duration was 23.42 s, and the average silence duration was 5.34 s (Table 2).

## 5. Analysis

### 5.1. Comparison of Number of Answer Words per Question

**Figure 4:**
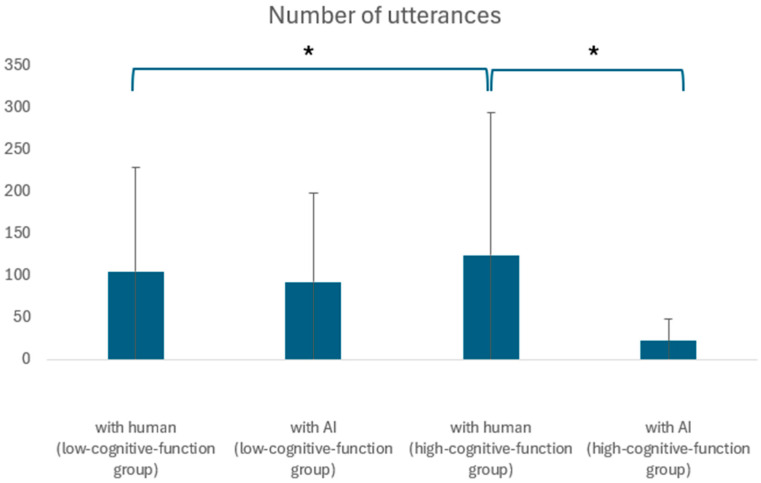
Comparison of high- and low-cognitive-function groups in terms of number of utterances. *: Significant differences were found at the 5% level.

The number of utterances was analyzed for its correlation with age and cognitive function using a two-tailed t-test for unequal variances at a significance level of 5%. No significant difference was observed across the entire dataset (*p* = 4.97, *t* = 5.04). Comparing conversations with AI and humans among participants from the senior talent center showed no significant differences (*p* = 3.09, *t* = 14.7). However, among the day service center participants, the average number of utterances in human conversations was significantly different from the AI conversations, being lower for humans (92.58) than for AI (104.88) (*p* = 0.05, *t* = −1.96). In conversations between AI and humans, a significant difference in the number of utterances was found between senior human resources and day service participants only in the AI conversations (*p* = 0.02, *t* = 2.28 for human; *p* = 1.75, *t* = −16.67 for AI). Figure 4 presents these results in a box plot format. Sections marked with a single asterisk indicate a significant difference at the 5% level, while sections marked with two asterisks indicate a significant difference at the 1% level.

### 5.2. Comparison of Speech Duration per Answer

The impact of age and cognitive function on speech duration was analyzed using a two-tailed *t*-test for unequal variances at a 5% level. No significant differences were found (*p* = 0.13, *t* = 1.53). Similarly, no significant differences were observed in speech duration between AI and human conversations among participants from either the senior talent center (*p* = 5.50, *t* = 10.07) or the day service center (*p* = 0.57, *t* = 0.57).

**Figure 5:**
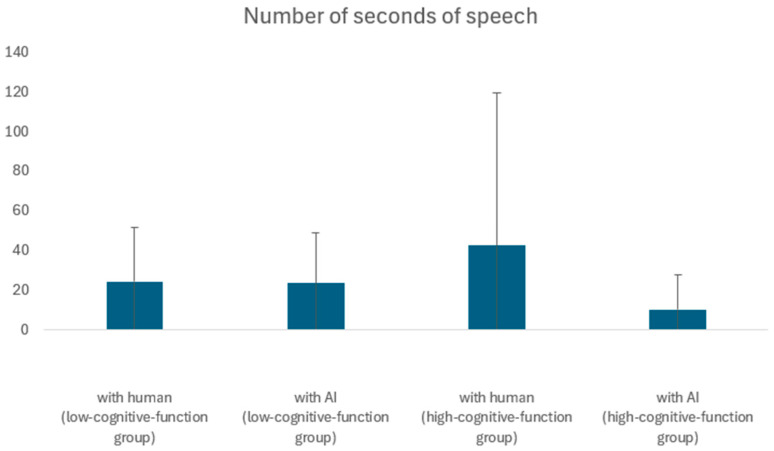
Comparison of high- and low-cognitive-function groups in terms of seconds of speech.

There was also no significant difference between the speech time of silver and day service participants when comparing the speech time of silver and day service participants when interacting with humans (*p* = 3.83, *t* = 5.56), and no significant difference was found between the speech time of silver and day service participants when interacting with AI (*p* = 9.80, *t* = −10.74). Figure 5 presents these results in a box plot format.

### 5.3. Comparison of Silence Duration per Answer

**Figure 6:**
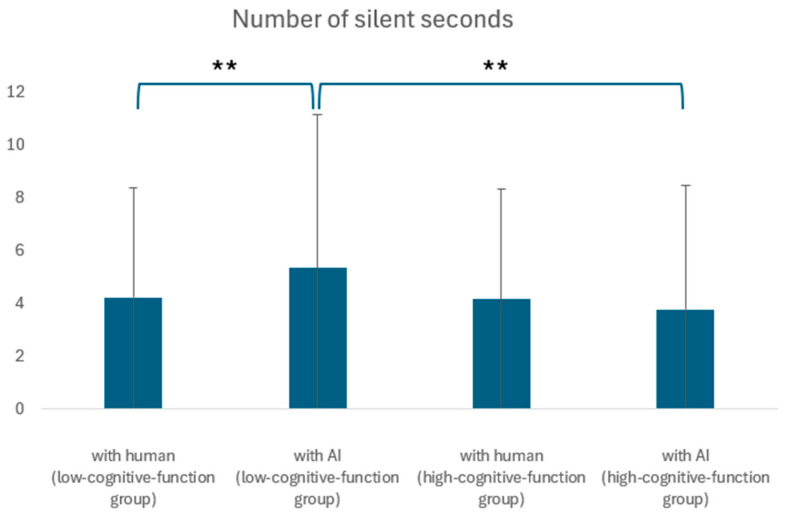
Comparison of high- and low-cognitive-function groups in terms of silent seconds. **: Significant differences were found at the 1% level.

Silence duration was analyzed for its correlation with age and cognitive function using a two-tailed *t*-test for unequal variances. A significant difference was found (*p* = 0.00, *t* = −2.80). In the senior talent center, no significant difference in terms of silence duration was observed between AI and human conversations (*p* = 0.44, *t* = 0.77). However, for the day service center participants, silence duration was significantly longer in AI conversations compared to human conversations, indicating possible discomfort or confusion among some elderly individuals when interacting with AI (*p* = 0.00, *t* = −3.05). Figure 6 presents these results in a box plot format. Within the group with cognitive decline, there was a significant difference in silence duration between the conversations with AI and the conversations with humans. Additionally, in the conversations with AI, there was a significant difference in silence duration between the high and low cognitive function groups. Sections marked with two asterisks indicate a significant difference at the 1% level.

## 6. Discussion

### 6.1. Effectiveness of AI Agents

This study confirmed that AI agents could be effective in engaging elderly individuals in conversation, particularly those with diminished cognitive functions. AI conversations resulted in a higher number of utterances compared to human conversations among participants from the day service center (*p* = 0.05, *t* = −1.96), suggesting that AI could facilitate dialogue under certain conditions equally well or better than humans. However, no significant differences in the number of utterances were observed among participants from the senior talent center (*p* = 3.09, *t* = 14.7), indicating that the effectiveness of AI may vary depending on the cognitive state of the participants. The increase in silence duration during AI conversations also deserves attention; it may indicate that AI conversations could impose cognitive or emotional burdens on some elderly individuals, necessitating further refinement in AI agent design to ensure psychological comfort. Additionally, research by Wang et al. (2022) has explored the potential of silence duration as a diagnostic biomarker for cognitive impairment, suggesting that if the differences in silence duration between human and AI conversations are mitigated, AI agents could contribute to the diagnosis of cognitive disorders [41].

### 6.2. Differences in Responses by Question Item

This study compared the seconds of speech and silence for each question item in conversations with AI and humans among participants from the day service center. A paired two-tailed *t*-test at a 5% level showed significant differences in speech seconds for questions about siblings (*p* = 0.00, *t* = 3.30), post-middle school activities (*p* = 0.00, *t* = 3.20), meal preparation (*p* = 0.04, *t* = −2.28), laundry (*p* = 0.02, *t* = −2.58), and respected individuals (*p* = 0.00, *t* = 3.50). The average speech duration was longer in human interactions for questions about siblings (40.65 s for humans vs. 13.00 s for AI), post-middle school activities (36.56 s for humans vs. 15.07 s for AI), and respected individuals (45.94 s for humans vs. 21.67 s for AI). These questions typically elicited responses related to memories or emotions. In contrast, questions about current daily activities such as meal preparation and laundry prompted longer responses in AI conversations, indicating the effectiveness of AI in facilitating dialogue on routine topics.

Cognitive fluctuations in dementia patients may affect the activities of daily living (ADL) and the quality of life [42], and the various challenges faced in the daily lives of dementia patients can create difficulties for both the patients themselves and their families [43]. As such, the assessment of ADL is considered highly important for rehabilitation and treatment [44].

A study by Yamada et al. (2021) found that assessing speech responses to questions about daily life using a tablet-based system could detect early signs of Alzheimer’s disease with accuracy, comparable to neuropsychological tests [45]. In our study, the trend indicating the effectiveness of AI in facilitating conversations about daily life suggests the potential use of AI in ADL assessment, providing new insights into the possibilities for utilizing AI in this area.

### 6.3. Effects of Age and Cognitive Function on Question Responses

Participants from the senior talent center, who generally had better cognitive function, showed no significant differences in speech duration or number of utterances between the AI and human conversations (*p* = 5.50, *t* = 10.07 for speech duration; *p* = 3.09, *t* = 14.7 for utterances), suggesting that AI agents can be as effective as humans in engaging cognitively intact elderly individuals. Similarly, no significant differences in silence duration were observed (*p* = 0.44, *t* = 0.77). On the other hand, participants from the day service center, who had lower cognitive functions, showed no significant differences in speech duration or number of utterances between AI and human conversations (*p* = 0.57, *t* = 0.57 for speech duration; *p* = 1.75, *t* = −16.67 for utterances). However, the increased silence duration in AI conversations suggests that AI interactions might be causing discomfort or confusion for these individuals, highlighting the need to customize AI dialogue systems based on individual cognitive abilities.

### 6.4. Future Work

Given that AI can elicit speech and speech duration at levels comparable to or better than human interactions, it stands as an effective dialogue method. However, the increased silence duration observed in AI conversations suggests room for improvement in AI’s questioning and response strategies. While age and cognitive function do not appear to significantly influence the number of words spoken or the duration of speech, they do affect silence duration, indicating the need for further exploration of factors that influence this aspect. Diederich et al. (2022) reviewed multiple studies on conversational agents. They highlighted that, due to advances in natural language processing, conversational agents are gaining increasing attention in both academia and practice. The authors emphasized the importance of designing conversational agents to meet their intended purposes and better understanding how humans interact with them. They also identified the need to accommodate diverse user groups, such as individuals with disabilities and the elderly, as a future challenge [46]. The fact that we were able to obtain quantitative data by examining the cognitive function of each interaction with a human or with an AI by dividing the cognitive function into groups will contribute to the development of AI agents for a variety of users in the future.

## 7. Limitations

This study conducted dialogue experiments based on a set of questions designed for routine conversation. Future research should explore the long-term effects of sustained interactions with AI to evaluate the sustainability and effectiveness of AI-supported communication aids.

In this study, the subjects were limited to elderly persons living in the community, and the conversation style was semi-structured interviews. Different results could be obtained with the elderly residing in nursing homes or hospitals or with a different conversation style. In Japan, it is difficult to select elderly people with cognitive decline as research subjects, and the sample size was small for this experiment. However, verification by prototype is necessary under such circumstances, and therein lies the contribution of this study. In the future, a follow-up study with a larger sample size is needed, using this study as a starting point.

In this study, the subjects were limited to elderly persons living in the community, and the conversation style was semi-structured interviews. Different results could be obtained if the elderly were residing in nursing homes or hospitals or if different conversation styles were used. In addition, although the MMSE was used for the cognitive function test in this study, different results may be indicated when different tests are used. In Japan, it is difficult to select elderly people with cognitive decline as research subjects, and the sample size in this experiment was small. However, even under such circumstances, verification by prototype is necessary, and this is where the contribution of this study lies. In the future, a follow-up study with a larger sample size is needed, using this study as a starting point.

## 8. Conclusions

This study examined the interaction dynamics of elderly participants during conversations mediated by AI versus those with humans. Key metrics, such as the number of utterances, speech duration, and silence duration, were recorded and analyzed to understand the impact of cognitive function and age on communication patterns.

The results indicated that while there were no significant differences in the overall number of utterances and speech duration between AI and human conversations, noticeable differences emerged when segmented by participant groups. Specifically, participants from the day service center exhibited significantly longer silence durations during AI-mediated conversations, suggesting a potential discomfort or difficulty in engaging with AI compared to human interlocutors. For participants from the senior talent center, the difference in interaction quality between AI and human conversations was minimal, indicating a potential adaptation or familiarity with technology. The absence of significant differences in utterance count and speech duration between AI and human interactions in this group supports the feasibility of using AI for routine conversational engagement with cognitively healthier elderly individuals.

The findings underscore the importance of tailoring AI-mediated interactions to accommodate varying levels of cognitive function and comfort with technology among elderly populations. Future research should focus on refining AI interaction protocols to enhance engagement and reduce discomfort, particularly for those with lower cognitive function or less familiarity with AI.

## Figures and Tables

**Table 1:** Daily conversation used in interpersonal and agent conversations, consisting of 30 questions across 5 areas (copyright Igarashi et al., 2023 [32]).

**(1) Process before coming to the hospital**
Q1. Where is your home?
Q2. How long did it take you to get here today?
Q3. After you left your home, how did you come here?
Q4. What time did you leave home to come to the hospital today?
**(2) Life history**
Q5. Where were you born?
Q6. Do you have any siblings (if so, how many)?
Q7. Which elementary school did you attend?
Q8. What did you do after elementary school? (Which junior high school did you attend?)
Q9. What did you do after graduating junior high school? (Which high school did you attend?)
Q10. What do you do for work? (Do you have any memorable stories?)
Q11. Are you married? (When was your wedding?)
Q12. Do you have any children? (Where do your children live?)
**(3) Normal life**
Q13. How do you usually spend your time? (Please tell us your approximate weekly schedule)
Q14. What time do you get up in the morning and go to bed?
Q15. How often do you go out? (Where do you go most often?)
Q16. Do you bathe every day? (Do you bathe in a bathtub?)
Q17. How do you prepare your meals? (Do you eat three meals a day?)/What did you eat last night?
Q18. How do you clean your house? (How often do you clean your house?)
Q19. How do you do your laundry? (How often do you do it?)
**(4) Interests**
Q20. What news have you been interested in on TV or the Internet recently?
Q21. Please tell me about a sad event that happened to you recently.
Q22. Please tell me about a recent unsettling event.
Q23. Tell me about a recent event that made you angry.
Q24. Tell me about a recent event that made you feel bad.
Q25. Tell me about a recent event that surprised you.
Q26. Tell me about a recent happy event that happened to you. When did it happen?
Q27. Tell me about someone you admire.
Q28. What are you passionate about these days?
**(5) Plans for the rest of the day**
Q29. What are your plans for the rest of the day? (How will you get home?)
Q30. When was the date of your last visit?

**Table 2:** Mean and SD for participants’ age, MMSE, number of answer words, speech time, and silent time.

				Number of Answer		Speech Time		Silent Time	
				Words Per Question		Per Question (s)		Per Question (s)	
		Age	MMSE	with Human	with AI	with Human	with AI	with Human	with AI
All	Mean	78.91	20.59	113.91	59.55	32.88	17.25	4.19	5.01
	SD	6.52	8.14	147.54	87.10	57.24	23.23	4.16	5.64
high-cognitive-function group	Mean	74.73	28.67	123.96	21.86	42.56	10.22	4.16	3.76
(Silver Talent Center)	SD	5.48	1.35	169.66	26.95	77.09	17.51	4.14	4.7
low-cognitive-function group	Mean	82.59	13.47	104.87	92.58	24.24	23.42	4.21	5.34
(Day Service Center)	SD	4.96	3.84	123.66	106.06	27.15	25.75	4.18	5.82

## Data Availability

Based on the requirements for the ethical review and the protocols outlined by our University for storing and sharing data, our data, which include information on individuals with dementia, will be disclosed upon reasonable request.
